# Sacrificial animals as evidence for the disintegration of the ritual and music system: A case study of the Zhuolu site (790-410 BC)

**DOI:** 10.1371/journal.pone.0318926

**Published:** 2025-02-12

**Authors:** Xin Yu, Hailin Liu, Zhe Zhang, Huiqiu Shao, Chunxue Wang, Dong Wei

**Affiliations:** 1 Bioarchaeology Laboratory in Jilin University, Changchun, Jilin Province, China; 2 School of Archaeology, Jilin University, Changchun, Jilin Province, China; 3 Department of History and Cultures, University of Bologna, Bologna, Italy; 4 JLU- SFU Joint Center for Bioarchaeological Research (JCBR), Vancouver, Canada; 5 Collaborative Research Centre for Archaeology of the Silk Roads, Northwest University, Xi’an, Shanxi Province, China; Universiti Teknologi Malaysia - Main Campus Skudai: Universiti Teknologi Malaysia, MALAYSIA

## Abstract

Ritual and music are important approaches to convey the ethical and moral concepts in the Zhou Dynasty (1046–256 BC) in China. The ritual and music (*liyue*) system, therefore, was established by the Duke of Zhou to maintain social order, create and strengthen the power of the Shang royalty. The essence of the system was the division of classes. The primary objective of this study was to analyze the sacrificial animals from the Zhuolu site (40°13´N, 115°24´E) in Hebei Province, China. We summarized the characteristics of sacrificial behavior during that period by examining the types of sacrificial animals and the specific parts of their bones. Ultimately, we discussed whether these characteristics align with the established rules of the ritual and music system. In this study, excavated animal bones were subjected to species identification, surface mark recording, and carbon and nitrogen stable isotope analyses. Skeletal surface marks and stable isotope analyses indicated that there are various methods by which people access and utilize animal resources. There are various animals, including pigs, deer, hares, ring-necked pheasants, and fish, were utilized in the burials. Included in these bones are not only the left and front parts of the body, but also the right part and the hind limbs. Not only is this inconsistent with the specified combination of animal species designated for sacrifice within the ritual and music system, but it also deviates from the requirement to utilize the left and front halves of the limbs. People may have changed and reduced the use of certain sacrificial animals according to their personal preferences and needs. This may be attributed to the disintegration of the ritual system during that period. By comparing different sites, it is possible to infer that the stringent execution of rules regarding the use of animals in burial rituals was directly related to the level of ritual system development during the period to which the site is associated.

## 1. Introduction

China is a highly ancient civilization with a lengthy and intricate history and culture. In recent years, Chinese archaeology has grown and numerous historical sites have been unearthed. Several large urban sites, such as Erlitou [1], Yanshi [2], Zhengzhou [3], and Anyang [4], have been found in the Central Plains where the Xia (2070–1600 BC) and Shang (1600–1046 BC) Dynasties are believed to have emerged [5]. The Zhou Dynasty, the third dynasty after Xia and Shang, was a period in which political systems, legal regulations and cultural customs gradually developed and formed a particular system. In order to maintain his right to rule, the *tianzi* (King of Zhou Dynasty) granted land as gifts to his relatives, meritorious officials, and the nobles of his ancestors, known as *zhuhou* (vassal kings). The *zhuhou* exercised relatively independent authority over their respective territories, but all owed allegiance to the king. The *zhuhou* distributed their lands among the *qingdafu* (ministers), who in turn allocated their lands to the *shi* (scholars). Consequently, a social hierarchy of ‘*tianzi*-*zhuhou*-*qingdafu*-*shi*’ was established [6]. The daily political and social activities of the ruling class were governed by rules and rituals, with specific music for these occasions, and separate rituals for different ranks of nobility, which were collectively known as the "ritual and music (*liyue* or ritual) system" [7]. It is not a mere hierarchy, but contains many cultural concepts, and similar to "religion"and "constitution", it is a kind of rule. *Li* is a term of considerable breadth that in different contexts may refer to specific rites and ceremonies, to the courtesies of social interaction, to an aspect of personal cultivation, to political and social institutions, and even to “culture” in the most general sense [8]. Music can add some interest to life, and it is responsible for creating a harmonious social atmosphere. The combination of the two made requirements and regulations for many things in people’s daily lives and became a means of control for the rulers. The ritual and music system dominated many elements of life at the time and is a crucial topic for archaeologists investigating human behavior and social interactions [9].

In ancient Chinese funerary practices, it was believed that after death, life in the netherworld was similar to life in this world. Consequently, the deceased were treated as if they were still alive, with their needs for clothing, food, shelter, and other aspects of daily life being adequately provided [10]. As a result, the size of tombs and the burial objects placed within them were closely aligned with the social status and rank of the occupant during their lifetime [11]. As time passed, the decay of rights led to a growing indifference toward ritual [12–14]. The ritual and music system imposed strict specifications for burial items, including containers, animals, and decorations within tombs [15]. The number and types of ancient relics, such as uncovered bronzes, decorative pieces, and tools, were favourably connected with the social standing of the inhabitants [16]. The type and number of animals buried can also reflect the social status of the tomb owner during their lifetime [17]. In the study of tombs from the Shang and Zhou periods, Su Xie and Xiangping Gao have thoroughly examined the sacrificial remains, attributing the similarities and differences in sacrificial practices to cultural and ethnic influences [18]. They focus on analyzing the relationship between the species, number, and orientation of sacrificed animals and the shape of the tombs, utilizing historical documents. Yanbo Song [19] and Zhipeng Li [20], through their observations of animal sacrifices in various Shang burials, reveal that people of that era preferred to use the forelimbs of animals in rituals and favored the left side for selection. Feng Luo has summarized the practice of animal sacrifice in Shang and Zhou tombs in northern China, noting that specific parts of the animal, such as the ribs and legs, were commonly sacrificed in the Yanshan region [18]. Funerary practices and characteristics, including the types and combinations of sacrificial animals, have been analyzed and summarized in several of the aforementioned studies at their respective sites. However, these studies do not explore the relationship between these features and the ritual and music system. The Zhuolu site is situated within the sphere of influence of the Yan culture. The remains discovered in its residential area indicate the advancement of animal husbandry during that period, while the animal bones found in the burial area hold significant value for understanding the funerary practices of the region. We have analyzed and documented the species, number, age at death, bone surface marks, and stable isotopes of animal bones excavated from burials at the Zhuolu site. Based on these data, we address the following questions: (1) What types of animals were sacrificed for burial, and which parts of their bodies were utilized? (2) What marks were present on the surface of the animal bones, and what human behaviors are associated with these marks? (3) How did people acquire these animals—through breeding or hunting? (4) What are the characteristics of the methods used for animal sacrifice? Do these characteristics align with the established rules of the ritual and music system? If not, what factors contributed to this discrepancy? Is there any additional evidence to support our findings? We aim to provide additional evidence and references for research on how people acquired and utilized animal resources during that period, as well as the relationship between the assemblage of martyred animals and the ritual and musical systems.

## 2. The archaeological background of Zhuolu site

**Fig 1:**
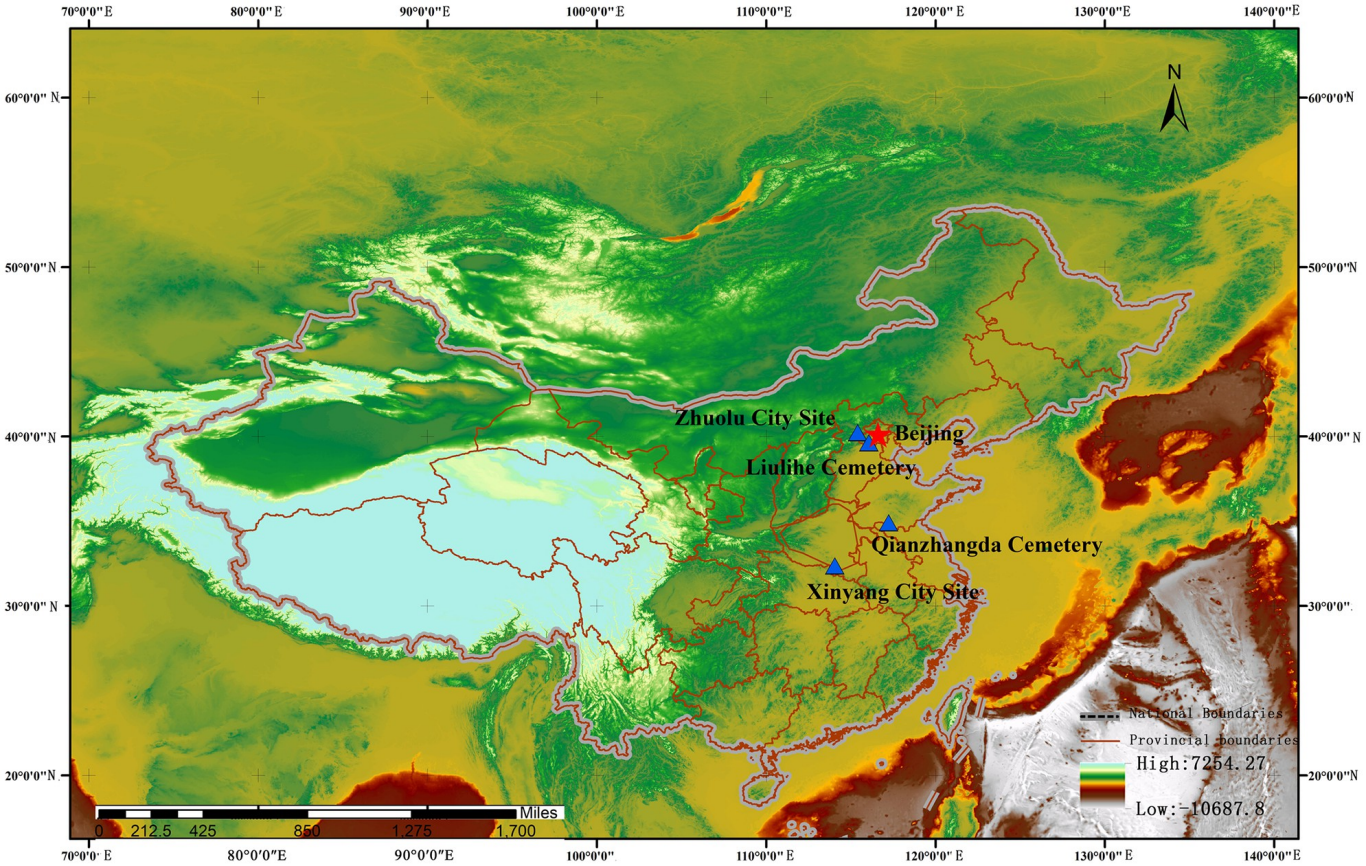
Location of Zhuolu sites. Images are quoted and modified from the paper illustrations. Base map from Openstreetmap with modifications (openstreetmap.org).

The Zhuolu site is situated 45 kilometers north of Sanbao Village in Fanshan Town and southeast of Zhuolu County in Zhangjiakou City, Hebei Province. The northeastern corner of the site is located at 40°13´N and 115°24´E, with a height of 733 meters above sea level at its center (Fig 1). The site is approximately 500 meters long from north to south, 500 meters wide at its widest point from east to west, and 450 meters wide at its narrowest point. The southern, northern, and western sides of the wall, which are surrounded by walls of varying heights, have been rather well preserved, while the eastern side has been completely destroyed by Lake Xuanyuan. A trial excavation was conducted in July 2017 by the Hebei Institute of Cultural Relics (now known as the Hebei Institute of Cultural Relics and Archaeology), the Research Center for Chinese Frontier Archaeological of Jilin University, and the Zhuolu County Cultural Relics Bureau to investigate the nature and chronology of the site. Nine explorations total, each measuring 5 m by 5 m, are positioned in the direction of 5°north by east in the excavation area, which is close to the central section of the western city wall. The overall excavation area is 225 square meters, and the thickness of the strata accumulation is 0.85 to 2.6 meters. The site contains pits, trenches, wells, tombs, and other relics. The uncovered artefacts include numerous tiles and bits of grey pottery, a few items made of stone, and animal remains. ^14^C dating results presumed that Zhuolu site was constructed during the Warring States period (770–476 BC) and was still in use during the Han Dynasty (202 BC—220 AD); however, the date of its abandonment is unknown [21]. Two tombs (designated M1 and M2), which are the main research features in this paper, that had been damaged by the planting of fruit trees and vines. They were founded along with pottery and bronze vessels, alongside animal sacrifice, were discovered during the excavations in the northern central area of the site.

**Fig 2:**
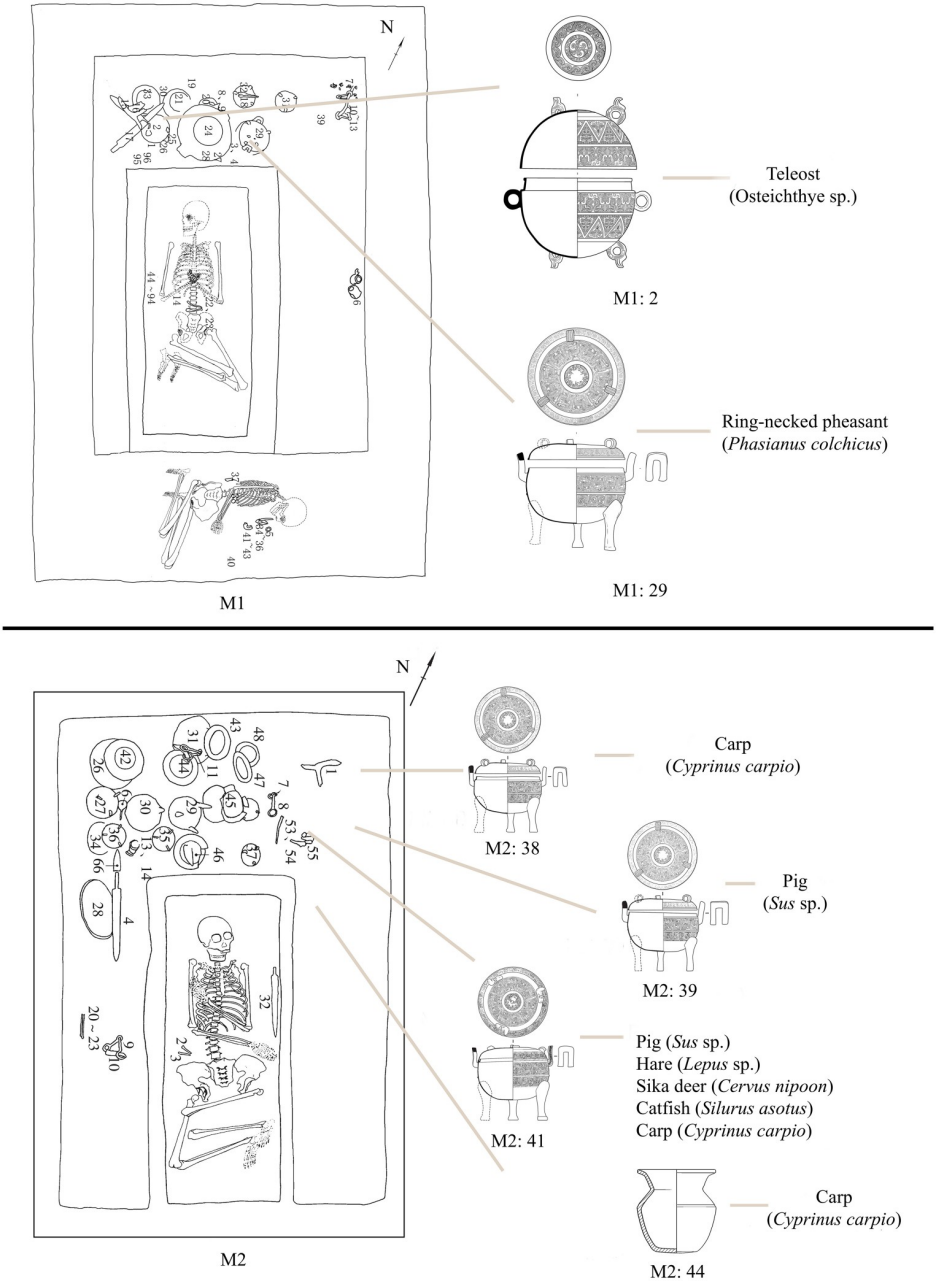
Sacrificial animals in burial containers.

M1 is a rectangular, vertical cairn tomb containing one inner coffin and one outer coffin, with the inner coffin resting on a coffin platform of sedentary soil (Fig 2). There is a second-tier platform of sedentary soil between the wall of the outer coffin and the tomb wall, with a deep niche inside the south wall of the chamber and above the second-tier platform. The tomb owner lay supine with his legs bent. The other, lying on his side with his legs bent, was buried on a second platform in the south of the chamber. A dog was placed between the coffins and outer coffins of the tomb’s owner to the northwest. Nine bronze vessels, including three tripods, two jars, one *dui* (a grain receptacle), one pedestal bowl, one plate, and one *yi* (gourd-shaped ladle or an ancient utensil for holding wine), are accompanied by six pottery vessels, including two pedestal bowls and two jars, one tripod, and one *zun* (a kind of wine vessel used in ancient times). In addition, there are numerous tools, chariot and horse fittings, and weapons with intricate designs [22].

M2 is a rectangular vertical pit. The remains of the tomb owner are found on the coffin bed, in a supine posture with bent legs (Fig 2). It was determined to be a man approximately 40 years old. The accompanying vessels include 12 bronze vessels (four tripods, two each of pedestal bowls, jars and *dui*, and one each of pedestal bowl and *yi*) and eight pottery (one duplex jar and seven *zun*). In addition, there are several implements, chariot and horse fittings, and bronze sacrificial ware. There is a groove in the bottom of the tomb to the north of the owner’s head. Tools, bronze vessels, containers and other objects were placed in the middle of this groove, while the decorative objects, weapons, chariot and horse fittings were primarily placed on the east and west sides of the coffin bed [23].

## 3. Materials and methods

### 3.1 Materials

The remains of 465 animal bones were found in the M1 and M2 burials, including a large number of fish fragments, a small number of mammal bones and very few bird bones. Some of the animal bones were found in the burial fill, and others were found in burial containers.

Bones of sacrificial animals were found in two burial artefacts in M1. A lot of fish (Osteichthyes) bones were cleaned up in No. M1:2 bronze *dui*, and some bird bones were found in No. M1:29 bronze tripod, which was initially identified as ring-necked pheasant (*Phasianus colchicus*). It should be emphasized that the dog bones excavated in the burial fill were not counted or described since they were poorly preserved and difficult to take out from tomb completely. In addition, the cowries (*Monetaria moneta*) are believed to have been buried as the decorative assemblage and are consequently excluded from this study.

Animal remains were discovered in the backfill and some burial artefacts (No. M1:2 bronze *dui* and No. M1:29 bronze tripod) of M1. After preliminary identification, these animal bones came from cowrie, fish, and ring-necked pheasants. The remains of animal bones such as domestic pigs (*Sus scrofa domestica*), sika deer (*Cervus nippon*), fish (Osteichthyes sp.), and hares (*Lepus* sp.) were found in the backfill and burial artefacts (No. M2: 38, 39, 41 bronze tripods and No. M2: 44 pottery *zun*) of M2 (Fig 2).

### 3.2 Methods

The animal bones covered in this study were collected by archaeologists from various parts of the site. The study did not involve any experiments on live animals and no animals were harmed. No permits were required for the described study, which complied with all relevant regulations.

#### 3.2.1 Data acquisition and statistics

The remains of sacrificial animals excavated from M1 and M2 were selected for collecting information of species, the number of identifiable specimens (NISP), the minimum number of individuals (MNI), and other pertinent data. Considering the unique characteristics of animal bones found in burials and the behavioral habits of people during that time, additional criteria were incorporated into the conventional method for calculating the number of the smallest individuals in this study. Firstly, each burial was counted separately, and the total was calculated afterward. Secondly, if an animal was discovered in different locations within the same burial, its number and orientation were recorded first. If the number of bones in each section is equal, it is necessary to determine whether they belong to the same individual based on the measurement data. For example, if the pig bones in tomb M2 are considered to represent one individual, then no hip bones were found associated with this individual. However, hip bones were discovered in bronze tripod M2:41, but the measurement data indicated that their articulation surfaces did not match in size to be joined, leading to their classification as two separate individuals.

Species identification of bones was conducted following this procedure: (1) Bone specimens were first cleaned, cataloged, and assigned their specimen numbers. (2) The anatomical position of each bone (e.g., scapula, humerus, ulna, radius, etc.) was assessed based on its morphology. (3) Interspecific differences in various anatomical features were carefully observed to determine the species of the bones (e.g., domestic pig, sika deer, sheep, etc.). The reference standards used were the atlas [24] and specimens from the Bioarchaeology Laboratory in Jilin University.

Measurement: The length and width of the bones were measured using vernier calipers (0.01mm), and data on their proximal, distal, and other features were recorded in accordance with established guideline [25].

#### 3.2.2 Age at death of animals

Since the skeleton excavated from the site lacked a skull, it is impossible to determine the age at death of the animal based on its teeth. In this study, the age at death of the animals is inferred from the healing of the epiphyses of their bones. According to Silver’s method for estimating the age at death of pigs, the epiphyses of the distal scapula, distal humerus, proximal radius, and hip bone are fully healed after 12 months of life [26]. Additionally, Carden’s study indicates that the proximal tibia of sika deer heals at an age exceeding 60 months [27].

#### 3.2.3 Observation of bone surface marks

The analysis of bone surface marks involves observing bone surface marks using magnifying glasses (30x) and handheld electron microscopes (10–200x) to record the frequency of distinct marks on different species of bones. The results of these observations are utilized to infer the cause of the marks and whether they were arranged as food, hence offering arguments for speculation about the sacrifices’ food role.

#### 3.2.4 Isotope analysis

Carbon and nitrogen stable isotope analyses are based on the composition of carbon and nitrogen isotopes in animal tissues to explain the consistency of the diet. According to the criteria of DeNiro [28] and Ambrose [29] for the determination of contamination, we selected 4 samples that were not contaminated by factors such as the buried environment and could represent the dietary information of the organism before death. The pre-treatment of bone collagen needed for the experiment was done at the Laboratory of Stable Isotope Chemistry, Institute of Vertebrate Paleontology and Paleoanthropology, Chinese Academy of Sciences. The surfaces of the bones were cleaned, and 2–3 g samples were obtained. Each sample was placed separately in a 50 ml beaker, to which 30 ml of 0.5 M HCl solution was added, and the samples were soaked at 4°C. The acid solution was replaced every two days. When the bone samples became soft and exhibited no obvious bubbles on their surfaces, they were washed with deionized water until a neutral pH was achieved. The neutral bone samples were then soaked in a 0.125 M NaOH solution for 20 hours. Afterward, the samples were washed with deionized water until they reached neutral pH levels. Subsequently, the neutral bone samples were soaked in a 0.001 M HCl solution at 70°C for 48 hours. The mixture was then filtered into a test tube while hot to obtain a collagen solution, which was subsequently placed in a freeze dryer for 48 hours to yield collagen.

The determination of collagen carbon (C) and nitrogen (N) isotopes was conducted in the stable isotope analysis laboratory at the Institute of Geology and Geophysics, Chinese Academy of Sciences. An elemental analyzer was coupled with a gas isotope mass spectrometer (EA-IRMS) to perform the tests. The Delta V model of the mass spectrometer was utilized. The organic carbon standards employed for determining carbon and nitrogen contents were graphite and urea, respectively, while glycine was used as the standard substance for nitrogen. During the sample testing process, one tin cup blank and one standard substance were included for every six sample test sequences. The standard substances used for carbon isotope measurements were GBW04407 (δ^13^C = −22.43%) and IVA urea (δ^13^C = −49.1%), while the standard substances for nitrogen isotope measurements were USGS64 (δ^15^N = 1.76%) and USGS65 (δ^15^N = 20.68%). The measured sample values were corrected using a two-point calibration method. The carbon values were expressed relative to the international standard VPDB, and the nitrogen values were expressed relative to the international standard Air-N2.

To analyze carbon, the photosynthetic route is divided into the C_3_, C_4_, and CAM pathways based on the CO_2_ fixation process in plants. The study of δ^13^C values in the bone collagen of humans and animals determines the type of food they consume [30]. Terrestrial wildlife has similar or identical recipe types and their primary food sources are C_3_ materials from their natural habitat [29], while the diet type of domestic animals is C_4_ [31]. The analysis of nitrogen isotopes is based on the enrichment of isotopes that exist as the trophic level rises. From the perspective of δ^15^N, the range of nutritional grades of herbivores is 3‰-7‰, and the range of nutritional grades of omnivores is 7‰-9‰ [32]. The measurement of stable isotopes of carbon and nitrogen permits the recovery, to variable degrees, of the food structure of ancient people and animals, leading to the investigation of topics linked to ancestral subsistence patterns and the domestication of animals.

## 4. Results

### 4.1 Skeletal parts used by the various sacrifice species

#### (1) Of the 465 bones found in these two burials, only 396 were identifiable

Among them were osteichthyes (Osteichthyes sp.), carp (*Cyprinus carpio*), catfish (*Silurus asotus*), ring-necked pheasant (*Phasianus colchicus*), sika deer (*Cervus nippon*), hares (*Lepus* sp.), domestic pigs (*Sus scrofa domestica*) and wild boars (*Sus scrofa*). Fish contained 59.1% of all recognizable specimens, followed by pigs with 21.6% (Table 1).

**Table 1:** NISP and MNI statistical table of animal remains.

Taxon	NISP	NISP%	MNI	MNI%	Discovered locations
Osteichthyes	36	58.8	1	8.3	M1: Bronze *dui*
Catfish	24	9.1	1	8.3	M2: Bronze tripod
Carp	174	6.1	3	25.0	M2: Bronze tripod; pottery *zun*
Domestic pig	70	17.7	3	25.0	M2: Bronze tripod
Wild boar	16	4.0	1	8.3	M2: burial filling
Hare	44	11.1	1	8.3	M1: Bronze tripod
Sika deer	4	1.0	1	8.3	M2: Bronze tripod, burial filling
Ring-necked pheasant	28	7.1	1	8.3	M2: Bronze tripod, burial filling

#### (2) Osteichthyes (Osteichthyes sp.)

**Fig 3:**
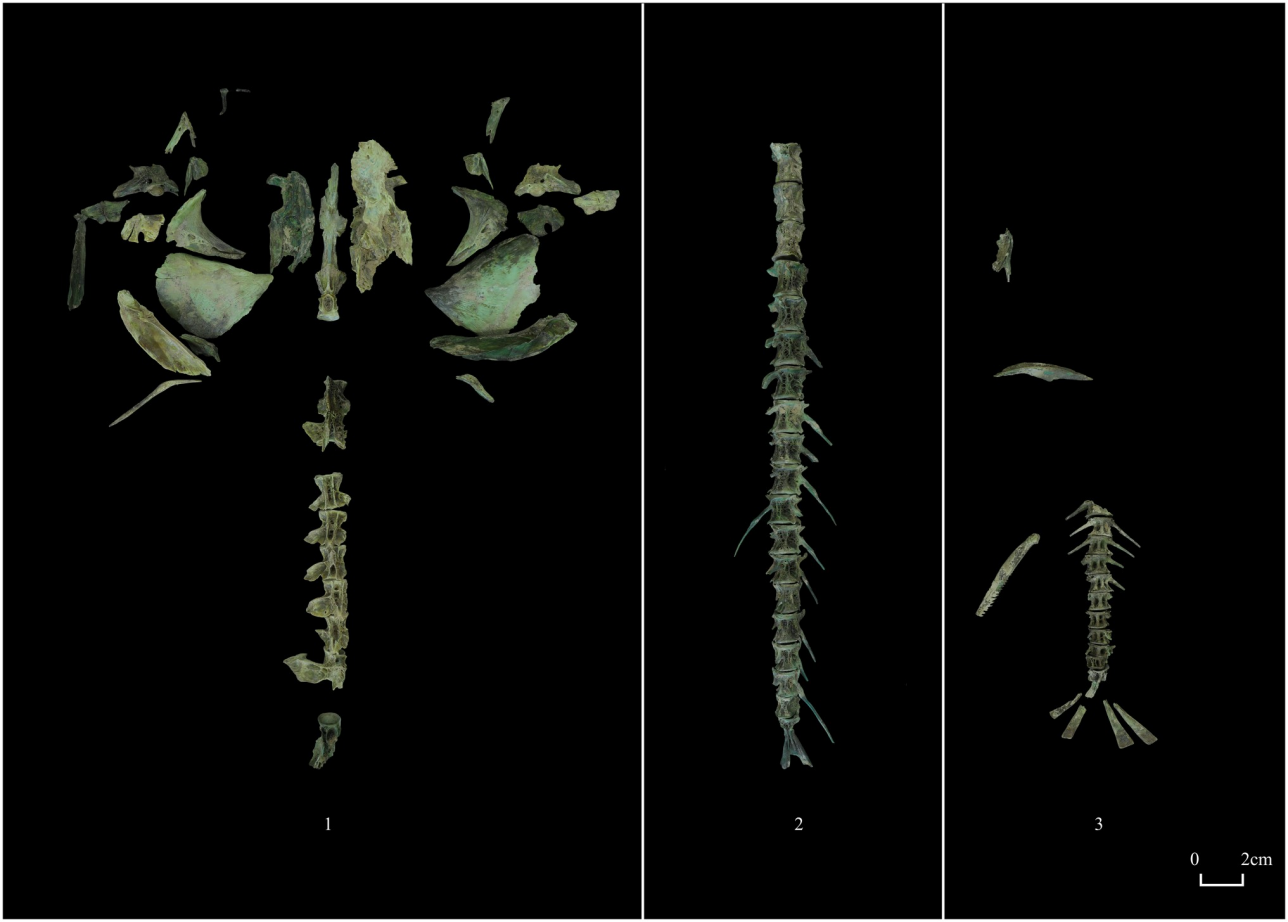
Fish bones. 1, Osteichthyes (M1: 2); 2, Catfish (M2: 41–55); 3, Carp (M2: 41–56).

There are 36 identified specimens, except for nine vertebrae, all of which are skulls. The operculum, cleithrum, frontal bones, parietal bones, horn bones, sphenotic bones, and pterotic bones have all been discovered. The structure of the individual’s operculum and vertebrae suggests that it was a carnivorous fish, but further identification of the species was impossible (Fig 3-1).

#### (3) Catfish (*Silurus asotus*)

Twenty-four specimens, comprising 19 vertebrae, two fin bones, and three ribs, were recognized, but no skull skeletons were found. These bones, with an MNI of 1, were discovered in M2 and should belong to the same individual (Fig 3-2).

#### (4) Carp (*Cyprinus carpio*)

Three containers with 174 recognizable specimens were discovered in M2. The bones were recognized and put together, and categorized based on their location within the dig. The specimens belonged to three individuals (designated I, II, and III) and the bones in the same vessel belonged to the same individual (Table 2). Carp I was discovered in the No. M2:38 bronze tripod with 99 recognizable specimens. There are 33 vertebrae, ten fin bones, and the remaining bones are skull skeletons. Carp II from the No. M2:41 bronze tripod includes 18 recognizable specimens, including two skulls, one spine, four caudal fin bones, and 11 vertebrae. This individual is poorly preserved, as just one left supracleithrum and one left alveolus survive from the skull. Carp III was found in the No. M2: 44 pottery *zun* containing 57 identifiable specimens. There are seven skulls, two spines, two medullary spines, twelve fin bones, and 34 vertebrae. The vertebrae of this individual are relatively well preserved. Bones from fish are less thick and osteoporotic. As a result of the flow of copper ions, the fish bones have taken on a darker copper-green hue (Fig 3-3).

**Table 2:** Fish bones existential records.

Skeleton	Carp I		Carp II		Carp III		Osteichthyes	
	L	R	L	R	L	R	L	R
Frontal bone	√	√						√
Operculum	√	√					√	√
Cleithrum	√	√			√		√	√
Suboperculum	√	√						
Interopercular bone	√	√						
Hypercleithrum		√	√				√	
Branchiostegal ray	√	√						
Postclavicle	√	√			√		√	√
Preoperculum	√	√					√	√
Coracoid	√	√				√		
Scapula	√	√				√		
Epihyal bone	√						√	√
Ceratohyal bone	√	√					√	√
Urohyal bone	√						√	
Hyomandibula	√	√					√	√
Maxilla	√	√						
Quadrate	√	√					√	√
Premaxillo	√						√	√
Lachrymal bone	√	√						
lachrymal bone	√	√						
lachrymal bone		√						
Infra-orbital	√	√						
Supra-orbital	√	√						
Orbitosphenoid	√	√						
Pterosphenoid	√	√					√	
Metapterygoid	√	√						
Sphenotic		√					√	
Ectethmoid		√						√
Pterotic	√							
Parietal bones		√						
Postfrontal		√						
Epiotic	√							
Exoccipital bone	√			√			√	√
Ethmoid bone	√							
Dentale	√		√				√	√
Hornbone							√	√
Supraoccipital bone		√						
Vomer bone	√							
Parasphenoid								√

Note: Skull parts not found in any of the above four individuals are not listed in the table.

#### (5) Ring-necked pheasant (*Phasianus colchicus*)

**Fig 4:**
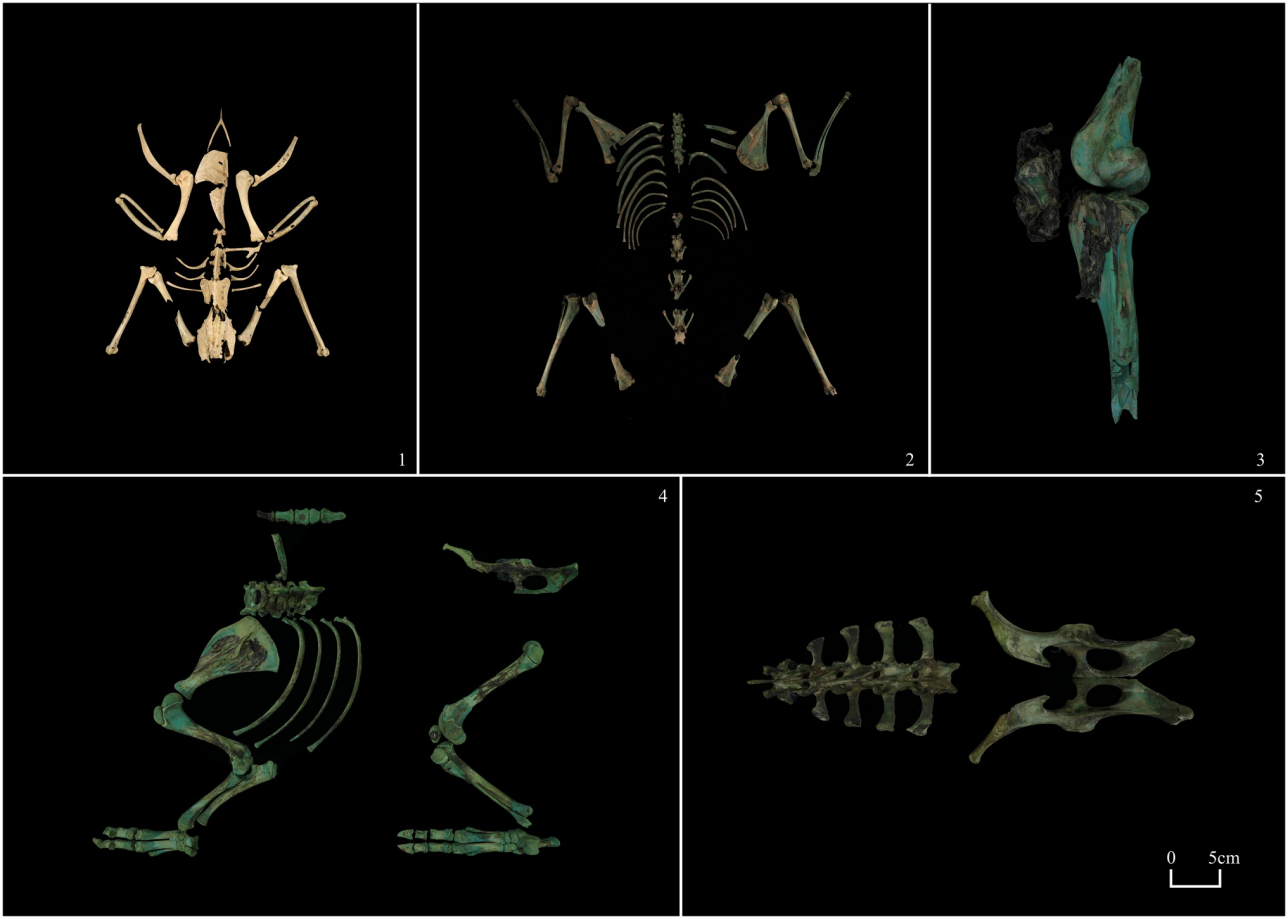
Animal bones. 1, Ring-necked pheasant (M1: 29); 2, Hare (M2: 41–4); 3, Sika deer (M2: 41–1); 4, Domestic pig (M2: 39–1); 5, Domestic pig (M2: 41–48).

Twenty-eight bone fragments were recovered, including two scapulae, two humeri, two ulnae, two radii, two tibiotarsus, one wishbone, one vertebra, seven ribs, one sternum, four femurs, two hip bones, and two keels. The femur, hip, and keel are incomplete and significantly fragmented. After rigorous examination and measurement, we conclude that these bones belong to the same individual (Fig 4-1). The healing of the epiphysis suggests that this is an adult individual. They are moderately deteriorated and in excellent condition of preservation, which even allows for the reconstruction of a nearly complete skeleton (Fig 4). Curiously, this skeleton lacks the skull and claw bones, and only one vertebra near the thorax was discovered. It is presumed that the ring-necked pheasant was not utilized entirely and that its claws, neck, and skull were removed for some reason.

#### (6) Hare (*Lepus* sp.)

Two scapulae, two tibiae, two humeri, four femurs, two ulnae, two radii, 13 vertebrae, and 17 ribs were retrieved from M2. Note that of the four humeral bones listed above, two are proximal, and two are distal and should belong to the left and right humerus, respectively; the 13 vertebrae consist of six thoracic and seven lumbar vertebrae (Fig 4-2). The healing of the epiphysis suggests that this is an adult individual. The bones above have a copper-green hue and are gently worn due to the bronze pigmentation. There are no neck and head bones, nor do they have limb terminal bones. The hare was also not used in its entirety, but rather the relatively anterior part of the carapace, excluding the head and neck, was chosen.

#### (7) Sika deer (*Cervus nippon*)

All four recognized examples are from M2, where archaeologists discovered a partially preserved antler on the left side of the burial backfill. We believe it is more likely a byproduct of horn ware production than the remains of a “sacrifice”. There was an incompletely preserved tibia with a fibula attached to its proximal end, the distal end of a femur, and a patella. The tibia, femur, and patella are all left-sided and are connected by tendons that have not been thoroughly corrupted (Fig 4-3). Consequently, their origin is established from the same body. The proximal tibial epiphysis had healed and the individual was older than 60 months at the time of death.

#### (8) Domestic pigs (*Sus scrofa domestica*)

**Fig 5:**
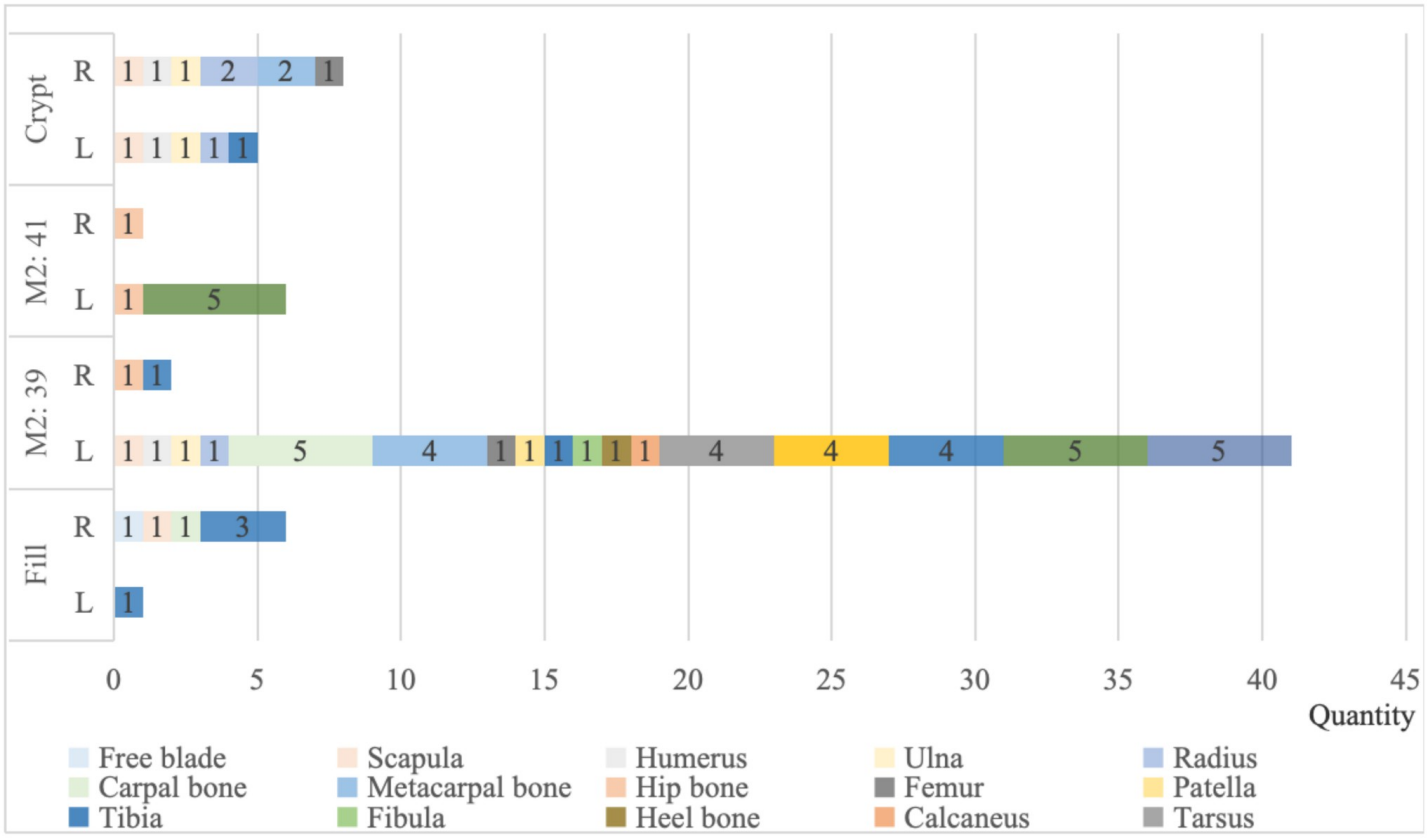
Statistics on the distribution location of domestic pig and wild boars bones (except sternum and phalanges).

Seventy identifiable specimens were recovered from the burial chamber and accompanying artefacts of M2. The skeletal parts found in these four places are both overlapping and different. The pig bones excavated from each burial container were counted separately, as described in Methods (Fig 5). We discovered that the pig bone in the No. M2: 39 bronze tripod preserves the left-sided portion of the limb, extending from the scapula to the forelimb, the four ribs, and the femur to the stem of the hind limb. The hip bone, however, is right-sided, distinguishing it from most left-sided bones. The seventh cervical vertebra and the third thoracic vertebra were also discovered (Fig 4-4). The healing of the distal scapular epiphysis and the humeral epiphysis indicates that the age at death for this individual was no more than 12 months. The No. M2: 41 bronze tripod contains a pig skeleton with a complete hip bone and five lumbar vertebrae attached to it. The preference of the side of the pig bones retrieved from the burial chamber is even more obscure. The bones from the scapula to the radius are bilateral, while two of the metacarpals and three of the forelimb limb bones are right-sided. The tibia is left-sided, although the femur is right-sided. The healing of the hip epiphyses shows that the age at death for this individual was no more than 12 months. Most of the skeletons discovered in the backfill were right-sided. Due to the complexity of the backfill, it is not employed as a research sample in this paper’s analysis. The lack of the skull and caudal vertebrae is a feature shared by the skeletal remains discovered in the areas mentioned above. This indicates that no pigs were utilized in their entirety.

#### (9) Wild boars (*Sus scrofa*)

A total of 16 boar bones were discovered in the eastern section of the chamber. There was one scapula, one humerus, one ulna, and one radius from both the left and right sides. Additionally, two metacarpals and three phalanges were found on the left side. The posterior half of the body contained one femur from the right side and one tibia from the left side. Measurements indicate that the bones originate from the same individual. The age of death was 1–2 years.

Based on the above, fish is used completely by people, while only certain parts of mammals are used. In this site, skulls and caudal vertebrae were not employed, and limb bones were utilized less frequently. For ring-necked pheasants and rabbits, the frequency of occurrence of the bones on their left and right sides is the same, and even the frequency of the bones on the anterior and posterior parts is very close. Deer were used for the left side of the hind limb bones. Additionally, people favoured the left or front part of the domestic pigs and wild boar carcass.

### 4.2 Surface modification of the bone

**Fig 6:**
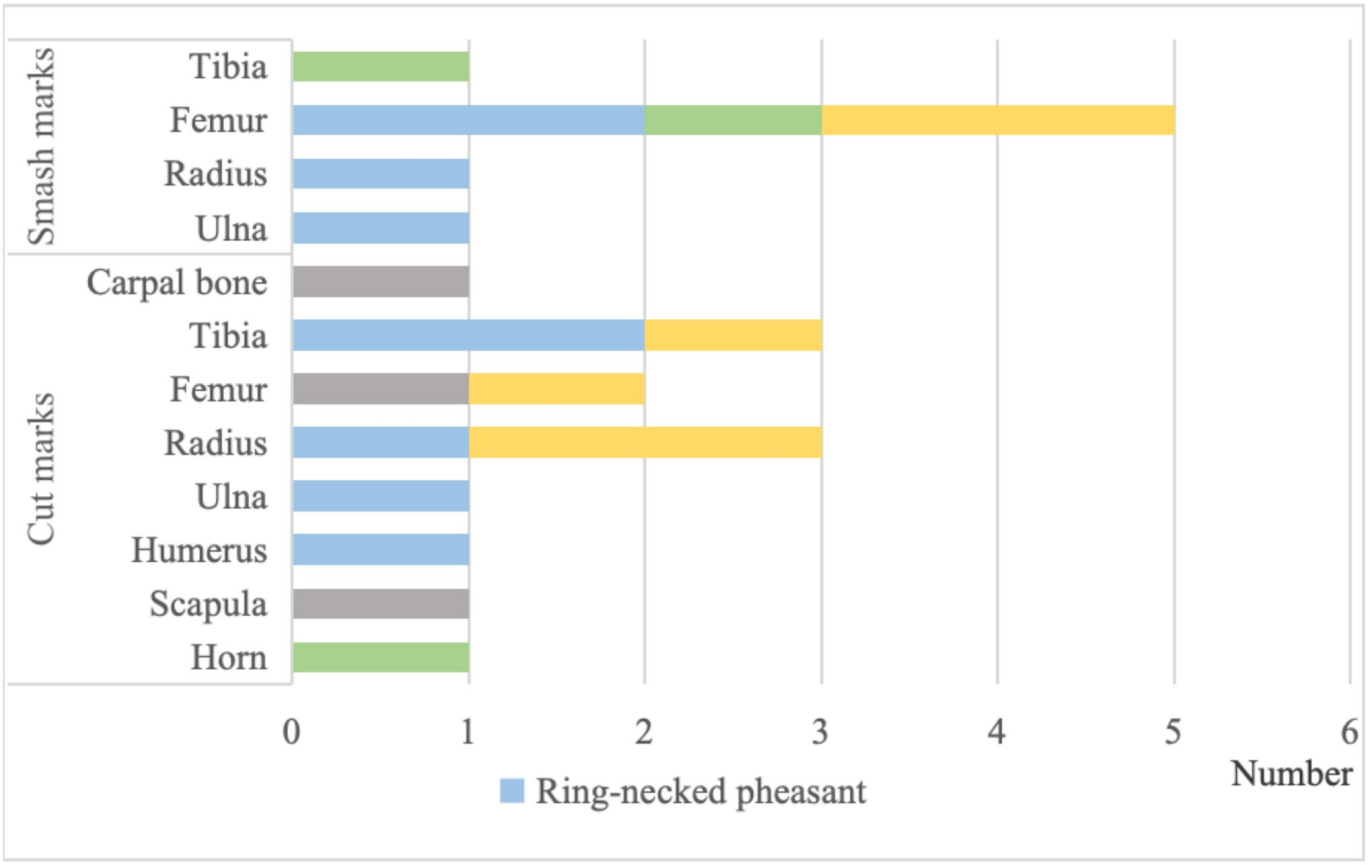
Statistics on the distribution of artificial marks on the bone surface.

**Fig 7:**
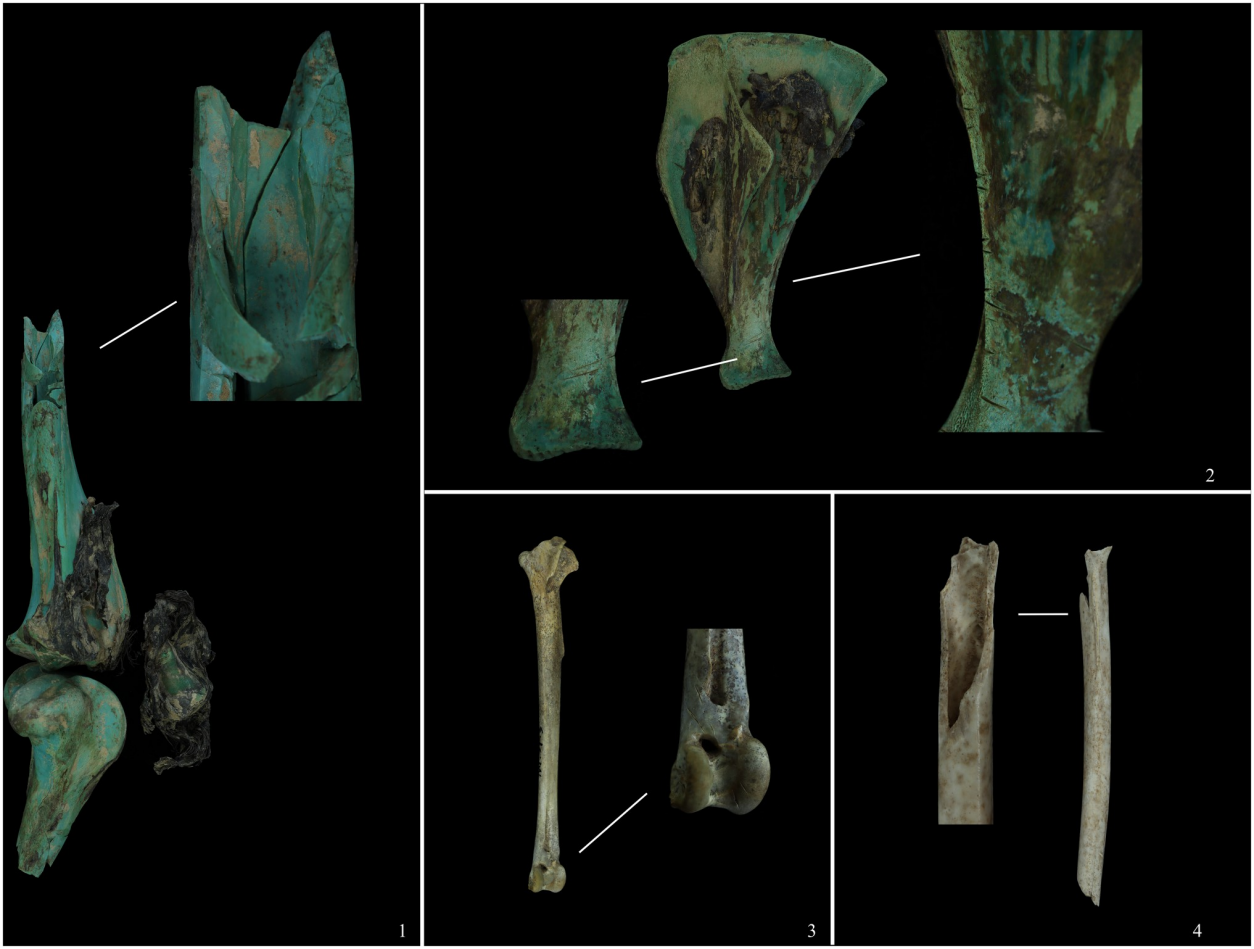
Bone surface marks. 1, Smash marks (deer); 2, Cut marks (domestic pig); 3, Cut marks (pheasant); 4, Smash marks (pheasant).

On the skeleton surfaces of these sacrificed animals, a total of 21 artificial marks were recovered. There are few types of marks, consisting primarily of cutting and smashing marks (Fig 6). The cut marks are generally short and deep, with a "V" shaped cross section. They are usually caused by a small force, when removing meat or dismembering. Smash marks generally have spiral or stepped fractures and are caused by larger forces (Fig 7). There are 13 cut marks, primarily at the joints of the bones of ring-necked pheasants, pigs, and hares, such as the proximal and distal ends. There are eight smash marks, primarily on the vertebrae of the limb bones of ring-necked pheasants, sika deer, and hares. Based on the position of the scars, it is indicated that they were left by inhabitants slaughtering the animal. Typically, the joints are simply covered by tendons or skin, making these areas easier to manipulate for dismembering. In contrast, the diaphyses is more robust and requires higher energy to sever or crush.

### 4.3 Analysis of stable isotopes

Despite the limited material, we still wanted to determine the source of sacrificial animals. For stable isotope studies, the researchers picked one wild boar bone, one pig bone, one hare bone, and one sika deer bone from M2 that were in good condition (Table 3).

**Table 3:** Experimental results of stable carbon and nitrogen isotopes.

No.	Taxon	Part	C (%)	N (%)	C/N	δ^13^ C ‰	δ^15^ N ‰
HDC1	Wild boar	Humerus	42.5	15.3	3.2	-21.94	2.02
HDC4	Domestic pig	Tibia	40.9	15.1	3.2	-8.06	8.01
HDC2	Sika deer	Limb bone	43.4	15.9	3.2	-19.89	4.18
HDC5	Hare	Femur	45.3	15.9	3.3	-19.64	3.45

As shown in Table 3, the δ^13^C values of the three samples—HDC1, HDC2, and HDC5—ranged from -30‰ to -22‰, indicating that their diets primarily consisted of C_3_ plants that were found in the natural environment surrounding the site. The δ^13^C values of HDC2 and HDC5 were similar, while HDC1 exhibited a more distinct value, likely due to the consumption of different types of C_3_ plants [33]. The δ13C value of HDC2 suggests that the plants in its diet were primarily C4 species. The significant presence of millet grains and other by-products in this individual’s diet is believed to be associated with the advancement of agriculture and increased food surpluses [34].

In terms of δ^15^N, the values for HDC1 are below the trophic level of phytophagous animals (3‰-7‰). The values for HDC2 and HDC5 fall within the trophic level of phytophagous animals, while the trophic level of HDC4 is situated within the range typical of omnivores (7‰-9‰) [32]. Trophic level enrichment indicates that HDC1, HDC2, and HDC5 are more closely associated with wildlife. Additionally, there were notable differences in the plant species present in their diets. In contrast, HDC4 exhibited a higher protein content in its diet, which may be linked to the composition of human food and feeding practices.

Combined with the anatomical morphology of the bones, it was determined that the individuals represented by HDC1, HDC2, and HDC5 were all wild animals, specifically wild boars, mergansers, and hares. The bones of wild boars were found in the burial chamber, while the bones of domestic pigs were contained in containers. This implies that there may be a difference in the nature of use between domestic pigs and wild boars, with domestic pigs having stronger food attributes. This may be tied to the tomb owner’s special status, indicating that he enjoyed hunting or a wild game throughout his lifetime.

## 5. Discussion

### 5.1 Characteristics of sacrificial animals at the Zhuolu site and its violation of regulations

#### 5.1.1 Types of sacrificial animals

A unique physician was in charge of and reconciled the king’s six types of meals, six kinds of drinks, six types of meat, numerous delicacies, various sauces, and eight sorts of delicacies. The phrase “six kinds of meat” refers to beef, lamb, pig, dog, goose, and fish [35]. Based on the ancient Chinese belief in the soul’s immortality, the living must treat the departed just as they did while alive, without disrespect. The “six kinds of meat” were a common part of people’s daily meals and were used in ceremonies and funerals. Utilization of the six animals (horses, oxen, sheep, pigs, dogs, and chicks) in late Shang Dynasty graves was already widespread. During this period, deer and rabbits were also essential commodities for sacrifice [36]. The usage of six animals at the Zhuolu site, among which pigs, pheasants, fish, and dogs, is consistent with the various animals chosen for sacrifice. The Zhou Dynasty maintained strict restrictions on the consumption and sacrifice of animals based on social status (Table 4). The classification of sacrificial animals and the manner in which they are organized in both M1 and M2 do not comply with the regulations. The specifications of the tombs and the burial objects suggest that the owners were local nobles, or at the least, members of the *qingdafu* class.

**Table 4:** The types of sacrifices to be used by each class.

Class	Taxon
*Tianzi*	cattle, sheep, pig, fish, cured meat, fowls and beasts, intestines and stomach, jerky, fresh fish,
*Zhuhou*	cattle, sheep, pig, fish, cured meat, fowls and beasts, intestines and stomach
*Qingdafu*	sheep, pig, fish, cured meat, fowls and beasts
*Shi*	pig, fish, cured meat

#### 5.1.2 Selection bias for bones

**Fig 8:**
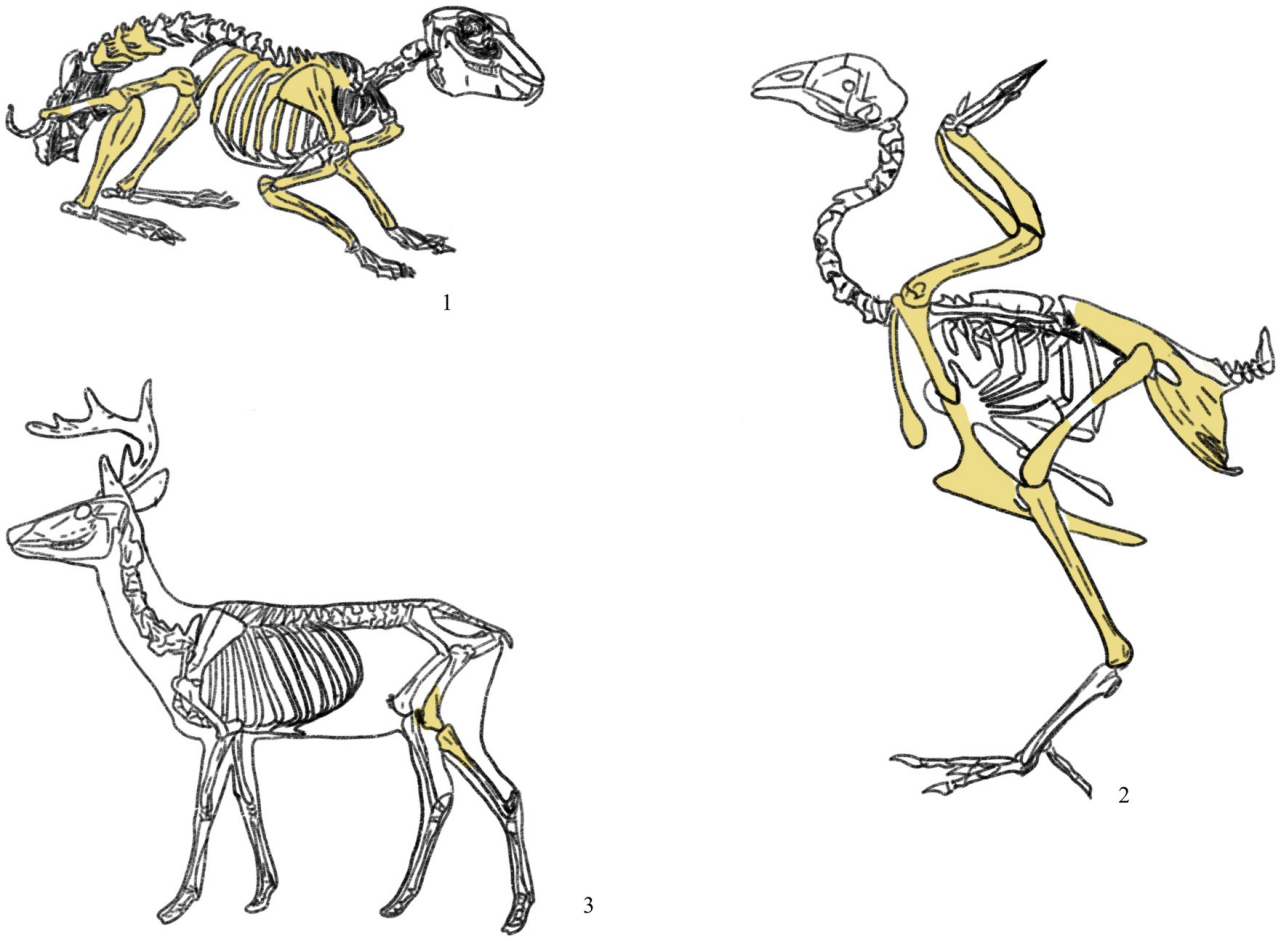
Schematic diagram of bone parts used as sacrificed animal in Zhuolu site. 1, Hare; 2, Pheasant; 3, Deer.

There were also clear rules about which limb parts of animal people chose to use as sacrifices. Additionally, the sections of the limbs were classified by their nobility. The upper portion of the hind legs was considered superior during the Shang Dynasty. In the sacrificial ceremonies of the Zhou Dynasty, it was believed that the front part of an animal’s body, particularly the shoulders, was more esteemed than the hindquarters [37]. When animals were sacrificed, their limb bones and ribs were frequently utilised, and people usually threw away the hoof parts of the limb bones [38]. Consistent with the preceding, no animal skulls were discovered at this burial site, and neither pheasant nor rabbit limb bones were utilized. Although people used the left limb of the deer, it was the limb bone at the back which did not reflect the characteristics of “more noble shoulders”. The use of pigs is very inconsistent with the regulations. Complete limb bones are present in M2:29, whereas the right side of the pig is used for the hoof. Overall, people employ a large number of forelimbs, but the likelihood of using the left or right side is roughly equal. The ancients believed hoof portions to be filthy. We observe that deer, pheasants, and hares were not employed, whereas pigs appear to have retained that element (Fig 8). This may be a result of changes in dietary choices. The hoof bones of the first three animals contained relatively little flesh and were, therefore, more likely to be removed, whereas those of the pig were edible and saved.

#### 5.1.3 Food processing

Researchers have found through comparative tests that boiling alters the organic composition of the bones and prevents lithogenesis [39]. S. J. Roberts boiled beef ribs in water continuously and recorded the condition of the bones after 3, 9, 27, and 81 hours. The longer the bone was cooked, the smoother its surface. But this variability needed more than 6 hours to be observed [40]. In the Zhou Dynasty, funerals involved animals that served not only as sacrificial offerings for the deceased but also as food for the participants in the rituals [41]. This dual role reflects the religious beliefs and cultural practices of society during that period. Some experts consider that the meat in the container was somewhat cooked. However, there is no in-depth research to corroborate this claim. The bones unearthed in the Zhuolu site are relatively light in weight. However, because the individual died at a young age and was affected by the burial environment, it is not certain that the bone loss was produced in the boiling process, so it cannot be effective evidence to confirm that the bones were cooked [42]. Fortunately, the position and frequency of marks such as slash marks and scratches can indicate one thing: at the time, the animals underwent preparatory food processing such as slaughtering, skinning, and dismemberment. Speculations on other findings will require additional investigation.

### 5.2 Other discrepancies with the ritual system

**Fig 9:**
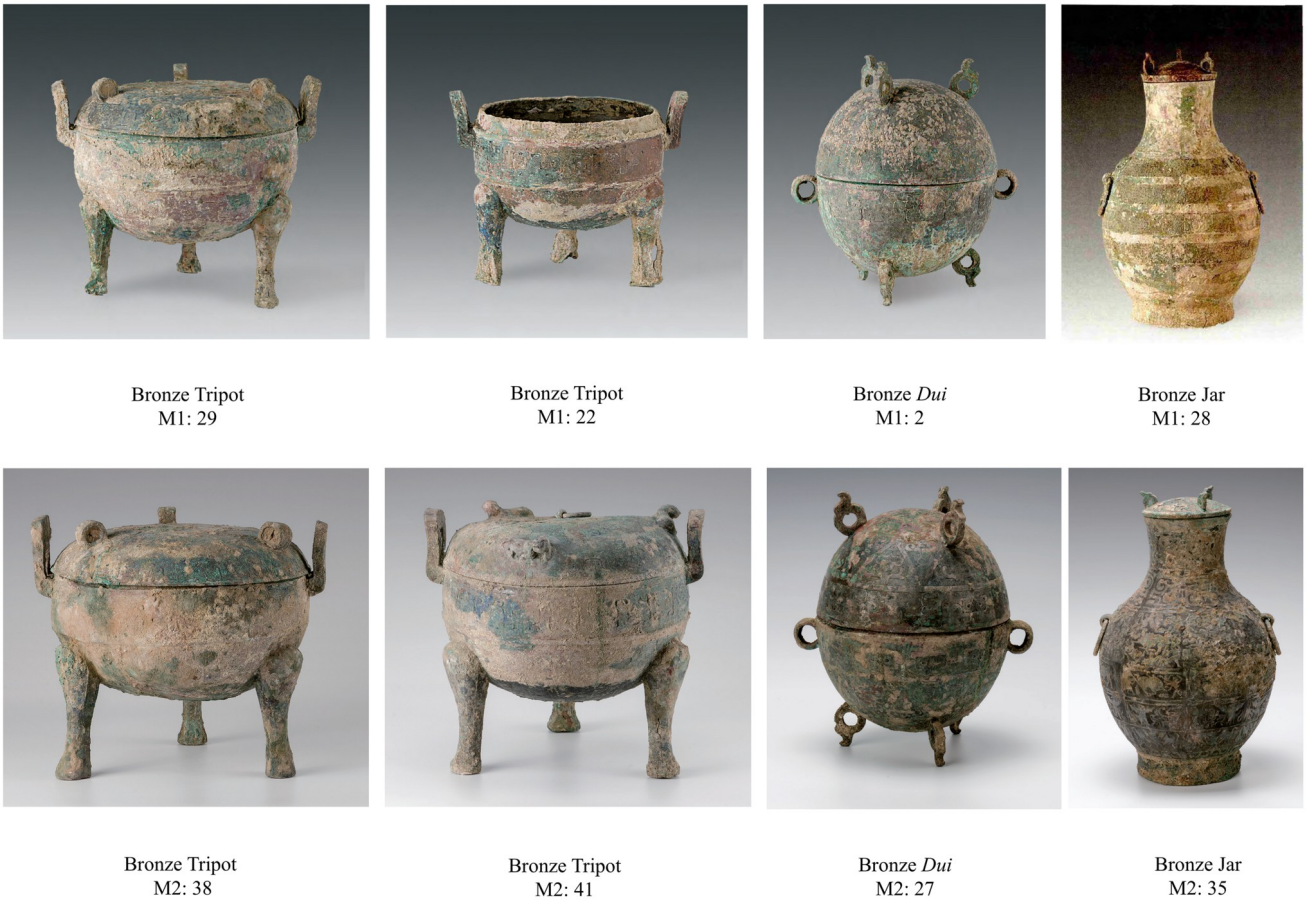
Photos of some buried artifacts.

Originally a common cooking utensil, the tripod was given a sacred meaning and became a ceremonial vessel over time because the meat of the animals in the rituals needed to be placed in the container. During the Zhou Dynasty, the number of tripods used by people was regulated according to their classes but was generally an odd number (Table 5). People in the Zhou Dynasty favored food vessels, and the combination of tripods and *gui* was central, with the number of *gui* generally one less than the number of tripods, and *dui* was commonly used instead of *gui* during the Warring States period. Five tripods are often found with jars, while three tripods are not accompanied by jars. In the Zhuolu site, three bronze tripods, one ceramic tripod, one bronze *dui*, two bronze jars and two ceramic jars were excavated from M1; four bronze tripods, two bronze *dui* and two bronze jars were excavated from M2 (Fig 9) No *gui* was found in either of these two burials, and the combination of other objects did not match the regulations. It should be emphasized that the use of earthenware ritual vessels instead of bronze ritual vessels is another indication of the decline of the ritual music system.

**Table 5:** Number of ritual wares used by each class (Unit: Piece).

Class	The number of tripods	The number of *gui*
*Tianzi*	9	8
*Zhuhou*	7	6
*Qingdafu*	5	4
*Shi*	3	2

### 5.3 The burials of animals at related sites

During the period from the end of the Western Zhou Dynasty (1046–771 BC) to the Spring and Autumn and Warring States Periods, the king constantly initiated wars against foreign tribes, while also intensifying repression and punishment of the domestic population. Such actions aroused strong discontent among the people. The failure of foreign wars led to a gradual weakening of the king’s military power, while the few successful wars increased the power of the vassals. The “favor for loyalty” exchange characterized the relationship between the Zhou king and the official aristocrats at the central court. With such a suicidal method of government in the “favor for loyalty” game, the Zhou king was inevitably moving to the losing side [43]. After the king’s power declined, he lacked effective central authority and lost his ability to control the vassals. The hierarchical order began to be disrupted as individual lords, like independent systemic units, gradually broke free from rituals to expand their territories or maintain their survival. The ritual system was eventually destroyed by a combination of political chaos, structural conflicts, and power confrontation.

Referring to factors such as the age and geographical location of Zhuolu site, we consulted the situation of sacrificial animals in the tombs of some sites (Fig 1). The first is the Qianzhangda cemetery (34°53´N, 117°12´E) from the late Shang Dynasty to the early Western Zhou Dynasty, which is located in Shandong Province. Bones of animals such as pigs, cattle, sheep, turtles, sika deer, bears, and fish have been excavated here. In addition, several mussel shells were discovered. For instance, the right forelimbs of pigs and sheep are in M21, but the left forelimbs of pigs, cattle, and sheep are in M38 [44]. The second is the Liulihe Yan cemetery (39°59´N, 116°02´E) in the Western Zhou Dynasty, which is a typical Yan culture cemetery in Hebei Province. The cemetery is dominated by slaughtered dogs, most of which are in fill or waist pits with their heads facing away from the tomb owner. It is not rare for sacrificial animals to have just partial skeletons; IM19 has a sheep leg bone placed between the burial materials, IM17 has a cattle leg placed on the burial objects, and M52 has a cattle skull in addition to a cattle leg [45]. Finally, there is the No.8 Tomb of Yangcheng Site in Xinyang City (30°27´N, 114°11´E), Henan Province, which is a remnant of the Warring States Period. Animal bones are primarily discovered within three pottery tripods and in the sediment surrounding one pottery pedestal. There are four types of animals: cattle, dogs, sheep, and pigs. The types of animals sacrificed for burial were determined by the status level (*Qingdafu*) of the tomb owner; however, the bones utilized did not adhere to the stipulations of the ritual and music system. The burial required the use of the animal’s left forelimb, left ribs, and the vertebrae from the front half of the body. A suckling pig needed to be relatively intact, excluding the head and hooves. In Tomb 8, however, the left and right sides of the cattle bones were found to be more evenly distributed, and hip and femur bones were also included. Suckling pigs were only represented by limb bones, with no bones from other parts of the animal. Only lumbar vertebrae from sheep were used [46].

During the Western Zhou period, when the ritual and music system was more flourishing, the types of animals buried in the tombs were the same as or richer than those specified. In addition to the animals specified in the ritual system, there may have been some other animals attached, which may also have been favoured by the tomb owner. People primarily utilized the forelimb and the left side of the animal for burials. During the Eastern Zhou Period (770–256 BC), when the ritual and music system declined, the types and parts of slaughtered animals were less extensive and less stringent than they had been previously.

## 6. Conclusion

The emergence and progress of ritual and music paralleled the development of human civilization. The evolution of the ritual and music system reached its zenith during the Western Zhou period. It was then severely impacted by the decline of royal power and the rise of the next class. It eventually collapsed utterly during the Spring and Autumn and Warring States periods. The same is true of the case study here. Not only do the assemblages of burial goods fail to convey the full splendour of the tomb’s occupant, but the types and portions of slaughtered animals sometimes conflict with the records. Although the concept and requirements of the ritual system were rigid and ordered, its practical application was ambiguous. Especially during social instability and conflict, it became more challenging to implement this spiritually demanding and complex burial structure. In our future research, we plan to correlate the study of animal bones excavated from the living quarters of the Zhuolu site with findings from the burial area to gain a deeper understanding of the role and significance of animals in ancient human societies. Additionally, we will contextualize the findings from the Zhuolu site within a broader geographic framework and systematically compile research on sacrificial animals from other sites in northern China. This comparative study will enrich our understanding of the relationship between sacrificial animals and the ritual and music systems, thereby providing new evidence and insights into the collapse of these systems. We anticipate that these studies will contribute valuable perspectives to the study of ancient societies and cultures.
